# How Do You Like Your Justice, Bent or Unbent?

**DOI:** 10.1515/mopp-2021-0072

**Published:** 2022-06-21

**Authors:** Lars J. K. Moen

**Affiliations:** University of Vienna, Vienna, Austria

**Keywords:** Estlund, justice, Rawls, utopophobia, verbal dispute

## Abstract

Principles of justice, David Estlund argues, cannot be falsified by people’s unwillingness to satisfy them. In his *Utopophobia*, Estlund rejects the view that justice must bend to human motivation to deliver practical implications for how institutions ought to function. In this paper, I argue that a substantive argument against such bending of justice principles must challenge the reasons for making these principles sensitive to motivational limitations. Estlund, however, provides no such challenge. His dispute with benders of justice is therefore a verbal one over the true meaning of justice, which need not worry those with the intuition that justice should perform a function that requires bending. By focusing on John Rawls’s reasons for bending his justice principles, I point towards a substantive critique of bent justice.

## 1 Introduction

A central claim in David Estlund’s *Utopophobia* is that principles of justice are not falsified by people’s unwillingness to satisfy them (Estlund 2020).1Henceforth, parenthetical page numbers will refer to pages in Estlund (2020). Justice does not bend to human motivation, Estlund argues. Justice must be sensitive to what people can do, but it need not be something we can realistically expect people to live up to. This might make the prospect of realising justice hopeless, but in Estlund’s view, a theory of justice that “requires more than we can ever expect to achieve is not thereby in the least flawed” (p. 4). “The fact that people will not live up to [the principles of justice] even though they could is, in that case, a defect of people, not of the theory”, he says (p. 84).

In this paper, I explore how Estlund’s rejection of bent justice principles challenges the position of justice benders. Proponents of bent justice clearly do not share, or at least give little weight to, Estlund’s intuition that justice is a moral standard we should not necessarily try to satisfy. They instead typically see justice as *the* value we should, all things considered, aim to realise. Rawls (1999a, p. 3) expresses this view when he famously states that “justice is the first virtue of social institutions, as truth is of systems of thought”. As we shall see, justice cannot perform its role as the first virtue unless it is bent.

Estlund finds “no adequate argument that the content of justice ought to defer to our proclivities” (p. 13). But I show that he does not substantively challenge reasons for bending. Estlund’s case against bent justice is therefore unlikely to move those who, like Rawls, give greater weight to the intuition that justice should serve a practical role that requires bending, and lesser weight to the conflicting intuition that justice is what it is whether realising it is compatible with existing motivational considerations or not. If we understand justice principles to serve a particular purpose in our theory, and they must be concessive to limits of human motivation to serve this purpose, then an argument against justice bending should include a rejection of its purpose.

A rejection of bent justice that does not challenge the reasons for justice bending risks amounting to no more than a verbal dispute over the true meaning of justice. In a verbal dispute, as Chalmers (2011, p. 515) notes, the parties are “not really disagreeing”.2Chalmers (2011) provides an insightful account of verbal and substantive disputes. For recent discussions of verbal disputes in political philosophy, see Bosworth (2020) and Moen (2022a). I elaborate on Chalmers’s distinction between verbal and substantive disagreements in Section 2. Estlund says he wants to avoid “semantic quibble” and “merely semantic” arguments (pp. 12–13). But a substantive, rather than merely verbal, debate with justice benders requires an argument for why justice cannot serve the purposes its benders attribute to it. Only then can we substantively argue that a principle of justice is not falsified by the hopelessness of motivating people to comply with them.

In Section 3, I explore Estlund’s view that principles of justice need play no practical role. Political philosophy, like mathematics, need not have a practical purpose, he says. But as will become clear in Section 4, Estlund’s rejection of practicalism and justice bending does not challenge Rawls’s reasons for making justice sensitive to motivational constraints. The tension between Estlund and justice benders therefore merely concerns what they take justice to be.

But I will not defend justice bending. In Section 4, I consider why Rawls makes the ideal of justice sensitive to facts about human motivation, and I identify grounds for doubting these reasons. Rawls bends justice to ensure it can serve as a realistic long-term target to pursue under real, non-ideal conditions. As many critics have pointed out, however, this use of an ideal is problematic. It is unclear how we can ever be confident a principle is appropriately bent to serve as a target for a large society to steer towards. It will also be difficult to design a plausible metric for assessing the progress towards the target. These concerns challenge such practical use of ideals in political philosophy. Ideals can still serve an evaluative purpose, but for that they need not be concessive to facts about human motivation.

These issues regarding the reasons for bending justice should be the focus in the debate over bent and unbent justice. With substantive arguments about the plausibility of these reasons, we avoid merely verbal disputes over the true meaning of justice.

## 2 Verbal Dispute

A verbal dispute concerns the language describing a certain domain. Unlike a substantive dispute, it is not about the facts within the domain, but the words used for describing them (Chalmers 2011, p. 515). Let us suppose two individuals, A and B, disagree about whether C’s statement, *x*, was a lie. To determine whether their dispute is verbal or substantive, we must ask A and B what they mean by “lie”. Suppose they both think it means “making a statement one believes to be false” and that they disagree about whether *x* was factually correct, or whether C believed it to be false. Their dispute then concerns the facts of the situation, not simply the meaning of “lie”. But suppose they agree about the facts—they both think C believed *x* to be true—and their disagreement is simply due to A’s understanding of “lie” as “making a false statement”, while B takes it to mean “making a false statement one believes to be false”. Their dispute is then merely verbal; it concerns the words describing the situation, not the facts about the situation.

In political philosophy, we might distinguish between the domains of fundamental principles and what G.A. Cohen (2008) calls “rules of regulation”. Facts about existing institutions and human motivation are excluded from the former, as fundamental principles are the basis for evaluating existing institutions. Such facts are crucial in the domain of rules of regulation, however, as these rules say how institutions actually ought to function given the existing social environment.

Cohen criticises Rawls for conflating these two categories by taking rules of regulation for fundamental principles. A consequence is that he loses a basis for criticising people for failing to meet basic principles, or for praising them for satisfying them. Cohen’s critique is not merely verbal, as it does not just say that Rawls uses incorrect terminology within a domain. He does not just say that what Rawls calls principles, we should call rules of regulation. That would be to disagree with Rawls merely about the terminology describing what Cohen calls rules of regulation. Cohen instead points out that Rawls fails to distinguish between two domains, which deprives him of resources for evaluating behaviour and institutions. Whether Cohen is right—whether there is a domain of more fundamental principles that is excluded from Rawls’s constructivist picture—is a separate issue I will not go into here.

## 3 Practicalism

Estlund also distinguishes between two domains in political philosophy: principles and proposals. A proposal, he says, is “a goal to set out for” (p. 10). It is necessarily concessive to facts about society, since what we should actually try to achieve depends on what people and institutions are actually like. Being unrealistic, he says, is a vice for proposals but not for principles (p. 26). Principles are not concessive, as they do not necessarily specify the right course of action. Concessive requirements, Estlund says, arise only from people’s failure to satisfy non-concessive principles (p. 31). Like Cohen (2008) then, Estlund sees principles as more fundamental than considerations of what ought to be done, all things considered, since such considerations must be sensitive to facts about how people actually are. On this view, there is no need to bend justice. Indeed, bending is impermissible.

But unlike Cohen, Estlund does not object to justice bending on the metaethical grounds that constructivism overlooks fundamental principles with an important evaluative purpose. He indeed suggests that Rawls can accept that there are more fundamental principles but then say that these are not the focus of his theory (pp. 176–177). Estlund’s argument is rather that justice should not be defined so that people are let off the hook just because they cannot be expected to live up to this ideal. In his example, Messy Bill keeps dumping his rubbish in the yard because he is too selfish and lazy to dispose of it appropriately (p. 28). In Estlund’s view, it would be absurd not to criticise Bill just because he lacks the motivation to act otherwise. As we have seen, failing to meet the ideal is, in Estlund’s view, “a defect of people, not of the theory” (p. 84). By taking this view, it is understandable that Estlund rejects what he calls ‘intellectual practicalism, or just “practicalism” for short, which is the view that “there is little or no value in studying or understanding anything unless this has practical implications” (p. 35).

Political philosophy, Estlund argues, need not have implications for political practice. At least in the domain of principles, there is no need to be concerned with practical purpose. Justice need not be something we should actually try to realise. While justice is an important value, we should not build the institutions it demands if we know people are very unlikely to comply with them (p. 117). A hopeless theory, he says, can be “merely evaluative” and need have no practical import (p. 118). We might have good reasons to forgo greater justice, though that would be regrettable (pp. 319–323). Estlund thinks reflection on social justice need not concern what should be done; it could involve nothing more than figuring out what to think about the matter of social justice (p. 61).

This rejection of practicalism leads Estlund to an analogy between political philosophy and mathematics. Practicalism cannot be true in pure mathematics, he says, since works in that discipline often lack any practical value (p. 310). Likewise, Estlund finds no reason for thinking practicalism is true in theorising principles of justice. “If you think math can have nonpractical value then you cannot deny a piece of political theory value on the bare ground that it has no practical value” (p. 310). He agrees with Cohen that some issues in political philosophy, including the evaluation of the truth of a theory of justice, are separate from practicalism and, in particular, the practicality of the principles of justice (p. 61).

Muldoon (2021, p. 595) challenges Estlund’s analogy between political philosophy and mathematics. The epistemic standing of proofs in pure mathematics, Muldoon says, relies on settled methods and definitions, but no such settlement exists in political philosophy. While some believe principles of justice need not have a practical role that requires bending, others believe they must serve such a practical purpose. Estlund may be right that practicalists have posed no successful argument against his non-practicalism (p. 308). But it is unclear why we should accept his contrary view that principles of justice need have no practical purpose and cannot be falsified by people’s unwillingness to satisfy them.

Returning to Estlund’s example, justice benders might agree that Bill’s behaviour is blameworthy, and they might also think just institutions can penalise Bill. But in cases where it would be less reasonable, for motivational or other reasons, to expect people to behave morally, one might not take justice to require such behaviour. In a world where most people are like Bill and institutional regulations are unlikely to improve their behaviour, we might concede that they violate no requirement of justice if we give justice a practical, regulatory role. Furthermore, if Bill really is unable to bring himself to dispose of the rubbish appropriately, Wiens (2016b, p. 338) argues that doing so is no demand of justice even for Estlund, given his commitment to the principle that ought implies can (pp. 26–29).

The point here, however, is that critics and defenders of bent justice have conflicting intuitions about how to describe a situation like that of Messy Bill, but this does not imply substantive disagreement. Benders can continue to maintain that justice must be sensitive to facts about human motivation because they understand justice to necessarily perform a practical role. A substantive critique of bent justice must include an argument against reasons for giving justice a practical role that requires bending.

## 4 Challenging Reasons for Justice Bending

We invite to a verbal dispute by merely saying the principles of justice need not have a practical purpose, as others can then simply respond by insisting that they must have such a purpose. We then just disagree about what terminology is appropriate for the things we talk about. A way of substantively rejecting practicalism and bent justice, on the other hand, would show that principles of justice cannot serve such a practical role. In this section, I consider Rawls’s view that justice must be bent to serve as a realistic long-term target. By showing why justice cannot be expected to serve this practical purpose, I challenge one influential reason for bending justice. While this is not a conclusive rejection of practicalism, as there might be other reasons for bending justice for some practical purpose, it is a substantive criticism of one influential view of why justice principles should be sensitive to facts about human motivation.

For Rawls (1999b, p. 90), the principles of justice as defined in ideal theory provide non-ideal theory with “an objective, an aim”. They are intended as a target to steer towards in non-ideal theorising. Zofia Stemplowska and Adam Swift (2012, p. 376) refer to this as ideal theory’s “target role”. As other defenders of this approach argue, we need an ideal to aim for, as we would otherwise pursue only small, short-term goods at the expense of opportunities to achieve larger, long-term benefits (Simmons 2010; Stemplowska 2008, pp. 332–334). This target role is an important reason for why Rawls’s principles are sensitive to human motivation. For the principles to serve as targets, it must be realistic to expect that people can bring themselves to comply with them. Challenging ideal theory’s ability to perform the target role is therefore to challenge a reason for making it sensitive to facts about human motivation.

Estlund suggests a similar role when he says justice enables us to think big, and he stresses that unforeseen moral progress sometimes occurs (ch. 13). But unlike Rawls, Estlund does not require that we can reasonably hope to satisfy the principles. He rejects what he calls “hopeful theory”, which is restricted so as to deliver nothing more than what we can reasonably hope to achieve (p. 119). His argument against bending here is that it prevents us from dreaming as big as we should. Such dreaming might lead to great improvements, though we need not have reasons to hope for it. Indeed, justice principles, in his view, remain intact even if we know they will not be satisfied (p. 84).

But Rawls and others who believe justice should perform a practical target role can allow people to dream hopelessly big while understanding the principles of justice to necessarily guide political practice and therefore be concessive. They can maintain that the principles must point towards realistic progress, which requires bending. Estlund’s intuition about justice conflicts with such a requirement, but Rawls and others can continue to give greater weight to the intuition that justice is a target we should actually aim for. When we have no realistic hope or expectation of people complying with the institutions required by justice, Estlund sees a concessive requirement not to build such institutions (pp. 30, 280). This does not affect the meaning of justice, in his view. It just means justice is not an appropriate practical goal (p. 83). This view conflicts with Rawls’s intuition that justice should serve the target role, but it is no challenge to Rawls’s reason for bending justice. It only tells us that Estlund and Rawls have conflicting intuitions about the meaning of justice.

Now, Estlund does make the substantive argument that Rawls’s principles of justice are unfit to perform the target role (p. 142). He bases this argument primarily on the observation that Rawls (2001, p. 13) defines his principles based on the unrealistic assumption that “(nearly) everyone strictly complies with … the principles of justice” (see also Rawls 1999a, pp. 7–8). For this reason, Estlund argues, the principles cannot serve a practical role under non-ideal conditions of partial compliance. Estlund thus joins a long list of critics objecting to Rawls’s full compliance assumption (Brennan and Pettit 2005, p. 258; Farrelly 2007, p. 845; Galston 2010, p. 405; Levy 2016, pp. 318–319; Schmidtz 2011, p. 778).3These critics do not consider how the full compliance assumption might perform a role in a model of fairness. Briefly, under ideal conditions of full compliance, every individual enjoys basic liberties and opportunities to pursue advantaged positions in one’s society in accordance with the principles of justice. Under non-ideal conditions of partial compliance, it would be unfair to deny anyone liberties and opportunities everyone would have had under ideal conditions even if that would improve the situation of individuals who are less well off in terms of these goods. This model of fairness can therefore work as a constraint on the pursuit of societal improvements (Moen 2022b, pp. 290–294). A similar ideal-theory based constraint is recognised by Korsgaard (1996, pp. 147–151), Schapiro (2003), and Taylor (2009).

But it is not quite clear how this is a criticism of ideal theory, since Rawls bends justice to make full, or nearly full, compliance realistic. The strains of commitment in his theory demand that compliance with just institutions cannot require compromising one’s fundamental interests (Rawls 1999a, pp. 153–160). And Rawls (1999a, pp. 105–109, 346, 398, 441, 509) further notes that principles that fail to attract widespread compliance are not the right principles for the particular society. Ideal theory is a precursor to non-ideal theory in the sense that the former gives the latter a target to aim for, but if we observe in our non-ideal theorising that the principles defined in ideal theory fail to serve the target role—if we see that they are hopeless—then they are not right for the society in focus, and we should go back to ideal theory and rethink the principles.

Herzog (2012) takes this to imply a “feedback loop” between ideal and non-ideal theory: ideal theory guides non-ideal theory, but non-ideal theory also informs ideal theory to ensure its practical applicability. This is apparent in Rawls’s later view that different societies can have different principles of justice. But some elements, and especially the principle demanding that everyone can effectively exercise the basic liberties, are uncompromisable (Rawls 2005, p. 450). Laura Valentini (2011, pp. 312–313) points out that critics have failed to appreciate this flexibility in Rawls’s theory.

The problem of second-best might give us one reason for rethinking the principles. Given the actual, non-ideal conditions, pursuing the ultimate ideal might lead to a state of affairs that is less attractive than the state of affairs we would realise by pursuing some other course of action (Brennan and Pettit 2005, pp. 260–263; Goodin 1995, pp. 52–55; Wiens 2012, pp. 55–56).4“The general theory of second best” was originally formulated by Lipsey and Lancaster (1956). For an analysis of its use in political philosophy, see Wiens (2016a). This is no problem for Estlund, since it might just mean the principles of unbent justice are hopeless and therefore no realistic target (ch. 14). But it is not necessarily a problem for the Rawlsian target role either, insofar as principles are bent so as to make the best state of affairs we can reasonably expect to achieve our target.

Estlund notes that such concessive theorising might be too lenient on obviously unjust practices, such as racism (pp. 23, 144). In a society with a large proportion of racist citizens, for example, equal civil rights might be successfully resisted however the state might act. Since we cannot expect the society to steer towards equal civil rights, this cannot be part of a conception of justice intended to perform the target role. Rawlsians might respond that their methodology and use of justice are not intended for just any society. Rawls (2005) himself, especially in his later work, notes that his theory is intended for liberal democracies, and one thing he presumably means is that such objectionable practices are not widespread in these societies.

This move also works as a response to Estlund’s objection that Rawls, in his political liberalism, bases the principles only on reasonable comprehensive doctrines rather than, in Estlund’s view, those more likely to exist (p. 142). He therefore cannot expect them to serve a practical role. This view seems peculiar, since the liberal values characteristic of a reasonable doctrine are widespread in a liberal democracy. Estlund, however, thinks such a society is unrealistic, and therefore seems less optimistic than Rawls does regarding people’s appreciation for equal enjoyment of the basic liberties in liberal democracies. I shall not try to measure such dispositions in these societies, and nor does Estlund. A more potent critical observation is perhaps that this leaves the theory with nothing to say about just institutions for societies that are not (yet) fully up to his standards of a liberal democracy (Scheffler 1994).

But again, there is no argument against the target role here. If justice benders keep clinging to the view that justice must be the value we should aim to realise, then they can continue to claim, contra Estlund, that justice principles should be adjusted in response to human motivation. For Estlund, such concessions do not affect the content of principles, but they do affect the content of proposals. Those who think principles are supposed to perform the target role, on the other hand, will object to this distinction. Estlund and these justice benders might therefore agree on how institutions should operate; they just disagree on whether to call the institutions just.

Showing why justice should not be bent for the purpose of the target role involves an argument against its ability to perform this role. To explore this issue, we must consider whether social institutions can realistically aim for a long-term target. This is a significant issue Rawls and other ideal guidance theorists have not done much to establish. Especially pressing is the concern that a theory providing a realistic long-term target depends on an analysis of causal mechanisms showing how we can reach it (Wiens 2012, pp. 63–64). Without such analysis, we cannot expect the principles of justice to serve their target role. And given the complexity of large, modern societies, it is reasonable to doubt that we will ever have such a causal theory.

A further problem for the target view of justice is based on the fact that a large society is not just complex but also continuously changing. What counts as a realistic target will therefore vary across time. As we keep learning new things about our ever-changing society, we must keep adjusting the target. To perform the target role, principles must be frequently adjusted and will therefore be a moving target rather than a fixed point to steer towards in the long term (Gaus 2016, pp. 59–61; Rosenberg 2016, pp. 63–65). With this need for continual revision, we can at no point confidently say that a principle is appropriately fact-sensitive to realistically guide long-term non-ideal theorising. We will have no grounds for saying that the principle can serve this practical target role.

Even if we can somehow fix justice as a realistic long-term target, another problem still remains. To say whether a move is taken towards the ideal or not, we need some way of measuring the extent to which different arrangements deviate from the ideal. For Rawls (1999a, p. 216; 2001, p. 13), institutional arrangements are more just the more they resemble perfect justice and, conversely, more unjust the more they deviate from this ideal. Estlund also finds potential value in using justice as a metric for comparing different alternatives (ch. 13). But unlike Rawls, he does not depend on such a measure to work out what ought to be done to move towards an ideal target.

Gaus (2016, pp. 42–43) doubts that such a measure is feasible. It would require giving a “justice score” to different institutional arrangements to assess how closely they resemble the ideal. But there are various ways of making such a measurement, and it is not clear which is most appropriate, or indeed whether any way is defensible. One arrangement can be closer to the ideal than another on one dimension but further away on another. Without good reasons for thinking one dimension more significant than another, we cannot say that one arrangement is more just than the other (Gaus 2016: 56–61).5This problem also applies to Estlund insofar as he maintains that justice can serve as a metric for assessing the justice of different arrangements (ch. 13). However, this point is of less importance for Estlund, as his view does not depend on justice performing this practical role.

Rawls (1999a, p. 216) might here turn to his lexical ordering of the principles of justice. Violations of the liberty principle are worse than any violation of the fair equality of opportunity principle, and violations of the difference principle are the least unjust violations of the justice principles. But as Sen (2009, p. 65) points out, it is implausible to give the liberty principle absolute priority (see also Hart 1973). Rawls also takes the priority rule to be too strict to apply under all non-ideal conditions. It is only “an approximate solution to the priority problem”, he says (Rawls 1999a, p. 39). But it is then unclear how to assess violations of the principles in terms of overall justice. The issue is complicated further when we account for other considerations, such as publicity, which requires that the public can see that fundamental institutions satisfy the principles of justice (Rawls 1999a, p. 154). It is unclear to what extent publicity matters if it conflicts with the most efficient ways of satisfying the principles. Simmons (2010, p. 18), who defends Rawlsian ideal guidance, also concedes that it is unclear how these different considerations are to be weighted against each other.

Now, critics of ideal guidance typically argue for more, not less, fact-sensitivity in political philosophy. We need to account for more facts to ensure our theorising can serve a practical purpose, and we should focus less on distant ideal principles and more on close, feasible improvements. But these arguments also imply that insofar as we think of justice as an ideal our society fails to live up to, and we cannot know how to make an ideal a realistic long-term target, then we cannot treat this ideal as a target. If the ideal cannot serve as a target, we have one less reason for making it fact-sensitive. Wiens (2012, p. 62, fn. 41) rejects ideal guidance, but he notes that his arguments do not count against Cohen’s fact-insensitive view of justice principles, as the principles can guide our attitudes towards institutional arrangements independently of any understanding of how to actually improve them. Sen (2009, p. 62) also notes that Cohen’s account of perfect justice might be right, but he adds that its insensitivity to real-world problems gives it little relevance for non-ideal theorising.

By rejecting the view that justice must be a target to aim for, we reject one reason for bending justice. Rawls makes principles sensitive to facts about human motivation to ensure they can constitute a realistic long-term target. But if we can at no point confidently say the principles are sufficiently fact-sensitive to serve this purpose, we have found a substantive argument against an influential reason for justice bending.

## 5 Conclusion

“There is no defect in a hopeless normative theory, and so none that hopeful theories avoid to their advantage”, Estlund says (p. 119). Rawls (1999a, p. 398), on the other hand, thinks principles of justice are “seriously defective” if they cannot attract the voluntary compliance of real people. In this paper, I have argued that a substantive, meaningful debate over these two conflicting views requires engagement with the reasons for seeing justice as sensitive to human motivation. Estlund insists that principles of justice are not falsified by people’s unwillingness to satisfy them. But unless we challenge the reasons why Rawls and other justice benders make the principles of justice sensitive to facts about motivation, we are left with no more than a verbal dispute over the meaning of justice.

Avoiding such debate over conflicting intuitions about terminology requires arguments against the reasons for justice bending. And I have shown that there are good reasons for doubting an important reason for bending justice. Rawls makes principles sensitive to facts about human motivation so that we can realistically expect people to, over time, be motivated to voluntarily comply with institutions that satisfy them. But it seems implausible that we can at any point say that a principle is appropriately fact-sensitive to serve as a long-term target. This argument, then, is a substantive challenge to justice bending.

The debate over principles of justice and whether they are falsified by people’s unwillingness to satisfy them should shift its focus away from a mere conflict of intuitions and towards the reasons for bending justice. Otherwise, we end up in a verbal dispute over the true meaning of justice, and out of such disagreement little progress can come (Chalmers 2015, p. 27).
